# Destruction of the Dorsal Motor Nucleus of the Vagus Aggravates Inflammation and Injury from Acid-Induced Acute Esophagitis in a Rat Model

**DOI:** 10.1155/2019/8243813

**Published:** 2019-06-04

**Authors:** Li Zhao, Pengyan Xie, Bin Geng, Zheng Wang, Le Xu

**Affiliations:** ^1^Department of Gastroenterology, Beijing Hospital, National Center of Gerontology, Beijing, China; ^2^Department of Gastroenterology, Peking University First Hospital, Beijing, China; ^3^Non-Coding RNA Medical Research Center, Peking University Health Science Center, Beijing, China; ^4^Pathology Department, Beijing Hospital, National Center of Gerontology, Beijing, China

## Abstract

**Background/Aims:**

The aim of this study is to examine the protective effect of the cholinergic anti-inflammatory pathway (CAP) in experimental esophagitis in rats.

**Methods:**

A total of 40 male Sprague-Dawley (SD) rats were randomly divided into five groups as follows: control group, sham + saline group, sham + acid group, operation + saline group, and operation + acid group. Two weeks after the dorsal motor nucleus of the vagus (DMV) destruction, hydrochloric acid with pepsin was perfused into the lower part of the esophagus for 90 min. The rats were sacrificed 60 min after perfusion. The esophagus was prepared for hematoxylin and eosin (HE) staining, and the degree of inflammation and NF-*κ*B activation in the esophagus was measured. Inflammatory cytokines (TNF-*α*, IL-6, IL-1*β*, and PGE2) in the esophagus were measured by ELISA. The brain was removed and processed for c-fos immunohistochemistry staining. The c-fos-positive neurons were counted and analyzed.

**Results:**

The TNF-*α*, IL-1*β*, IL-6, and PGE2 concentrations in the esophageal tissue increased after acid perfusion. The microscopic esophagitis scores and the activation of NF-*κ*B p65 in the esophagus were significantly higher in the operation + acid group than in the operation + saline group. c-fos-positive neurons significantly increased in rats receiving acid perfusion in the amygdala (AM), the paraventricular nucleus of the hypothalamus (PVN), the parabrachial nucleus (PBN), the nucleus of the solitary tract (NTS)/DMV, the nucleus ambiguous (NA), the reticular nucleus of the medulla (RNM), and the area postrema (AP). After DMV destruction, c-fos expression was reduced in the AM, PVN, PBN, NTS/DMV, NA, RNM, and AP, especially in the AM, PVN, NTS/DMV, RNM, and AP.

**Conclusions:**

The DMV is an important nucleus of the CAP. The DMV lesion can aggravate esophageal inflammation and injury from acid-induced acute esophagitis in a rat model. The CAP has a protective effect on the acute esophagitis rat model and could be a new therapy for reflux esophagitis (RE).

## 1. Introduction

RE is one type of gastroesophageal reflux disease (GERD). The prevalence of esophagitis has increased in recent years, with a range from 6.4% in China to 15.5% in Sweden according to population-based studies [1, 2]. RE is characterized by esophageal motility disturbances and the upregulation of a variety of inflammatory cytokines in the esophagus [3]. The high prevalence of GERD and its troublesome symptoms pose significant societal consequences and adversely impact the quality of life of individual patients [2]. Recently, the immunomodulatory function of the CAP has attracted experts' attention in studies of anti-inflammatory responses in animal disease models [4, 5]. However, the regulatory mechanism of the CAP in RE is still not clear. The CAP released acetylcholine (Ach) through vagal efferent fibres, and some studies have indicated that this anti-inflammatory effect was mediated by the interaction of Ach with nicotinic receptors on macrophages, which led to inhibition of macrophage activation and decreased cytokine production [6]. This so-called CAP may represent a regulatory system controlling the inflammatory response to a wide range of threats to the organism. Mechanical and chemical signals are transmitted to NTS via the vagal afferent fibres. After integration of the incoming information through neuronal communication between the NTS and DMV, vagal efferent fibres originated from the DMV are triggered to adjust gastrointestinal motility [7]. The presence of this feedback loop may represent an interesting mechanism controlling inflammation in a number of disorders. In contrast to hormonal control by corticosteroids, control via the autonomic nervous system may provide a rapid and target-specific response [8].

The DMV is an important visceral motor nucleus. Previous research showed that the rostral and caudal portions of the DMV contained esophageal preganglionic neurons. The esophagus and the lower esophageal sphincter (LES) receive inhibitory input from the caudal neurons and excitatory input from the rostral neurons [9]. Previous experiments by our group have shown that c-fos expression significantly increased in the NTS/DMV and AP in rats receiving an acid plus pepsin perfusion [10]. The proposed mechanism was that exposure of the lower part of the esophagus to acid-pepsin stimulated the mucosal receptors, which in turn activated the neurons of the NTS and then of the DMV to modulate esophageal peristalsis [11].

To investigate the protective effect of the CAP in an acute model of esophagitis in rats, an acute esophagitis rat model was created by unilateral DMV destruction and esophageal acid instillation. The presence of inflammatory lesions, the expression of inflammatory cytokine, and NF-*κ*B p65 activation in esophageal tissue, besides the c-fos expression in the brain nuclei, were analyzed.

## 2. Materials and Methods

### 2.1. Animals

Forty male SD rats weighing between 280 and 350 g were housed in standard home cages under conditions of controlled illumination (12 : 12 h light/dark cycle), humidity, and temperature (19-26°C) for at least 7 d prior to the experimental procedure. The rats were fed a standard rat diet and tap water. Standard laboratory chow was withdrawn 16 h before handling, but free access to water was maintained. All experimental protocols were approved by the Committee for Animal Care and Usage for Research and Education of Peking University First Hospital, and the permit number was J201204.

### 2.2. Experimental Methods

The rats were randomly divided into five groups as follows: a control group (*n* = 8), a sham + saline group (*n* = 8), a sham + acid group (*n* = 8), an operation + saline group (*n* = 8), and an operation + acid group (*n* = 8). The control group did not receive any stimulation. In the sham-operated and saline/acid groups, an electrode without a stimulator was inserted vertically into the right side of the brain stem at a level of 13.24 mm caudal to the Bregma and 0.7 mm lateral to the midline with a depth of 8.15 mm below the dorsal surface, and the saline/acid solution was perfused into the esophagus 2 wks later. In the operation and saline/acid groups, unilateral DMV destruction was performed, and the saline/acid solution was perfused into the esophagus 2 wks later.

### 2.3. Approach for Unilateral DMV Destruction

The rats were intra-abdominally anesthetized with urethane (1 g/kg) (Sinopharm Chemical Reagent Beijing Co. Ltd., China) and placed in a SR-5N stereotaxic apparatus (NARISHIGE GROUP Company Profile, Japan). The dorsal surface of the brain stem was exposed by limited occipital craniotomy. According to the coordination of the DMV as defined by the atlas, a monopolar Ni Cr alloy electrode (0.2-0.3 mm tip diameter) was inserted vertically into the right side of the brain stem at a level of 13.24 mm caudal to the Bregma and 0.7 mm lateral to the midline with a depth of 8.15 mm below the dorsal surface. Stimulation was provided by the RM6240C biological experimental system (Chengdu Company, China). An electric current (1 mA, 0.2 ms, 10 Hz) was passed through the DMV for 30-45 seconds [12].

**Figure 1:**
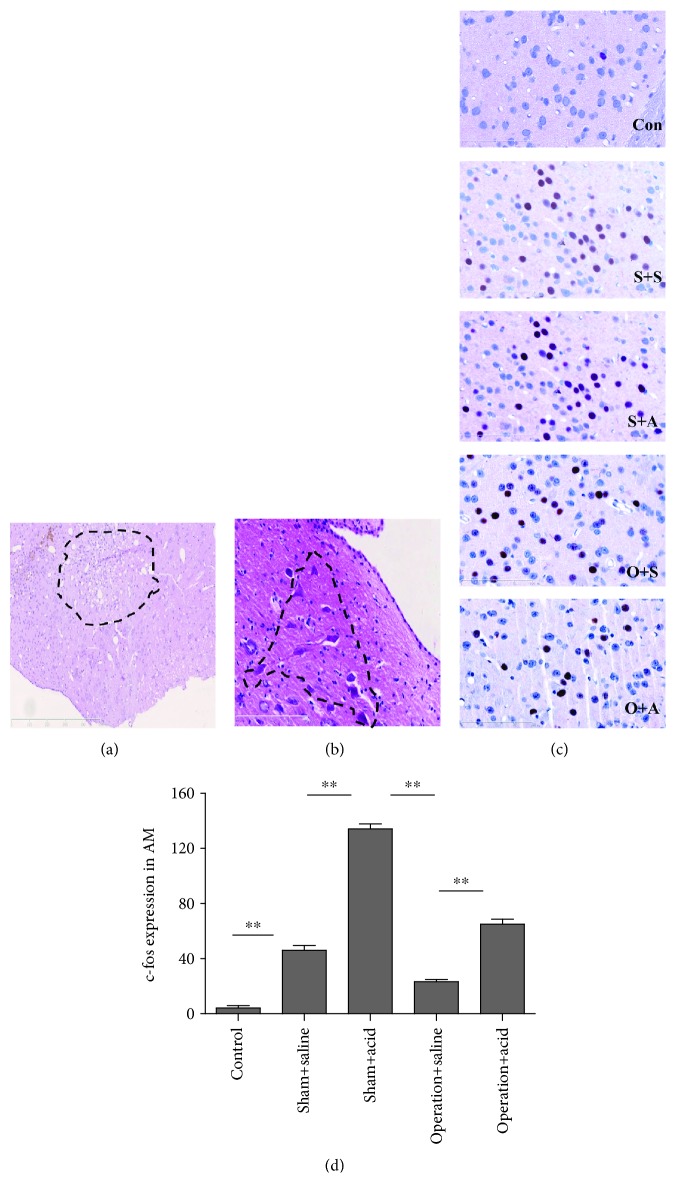
(a) Two weeks after DMV destruction under a light microscope: concurrent with axonal degeneration, myelin was disintegrated, which resulted in lipid and neutral fat, stained red-stained with Sultan III, activated microglia changed into phagocytic cells, necrotic neurons were devoured, bubble cells appeared, astrocytes proliferated, and ultimately honeycomb glial scars appeared. (b) Twenty-four hours after DMV destruction under a light microscope: the nuclei of nerve cells presented with pyknosis, the cells shrank and were deformed, Nissl bodies of the cytoplasm dissolved and disappeared, and then the cells dissolved and disappeared, creating so-called ghost cells that were deeply red-stained by HE. (c) c-fos-positive cells with red nuclei in the AM of all animals. Con: control group (×400); S+S: sham + saline group (×400); S+A: sham + acid group (×400); O+S: operation + saline group (×400); O+A: operation + acid group (×400). (d) c-fos expression in the AM increased more significantly in the sham + acid group than in the sham + saline group (*P* < 0.01). After DMV destruction, c-fos expression more significantly decreased in the AM in the operation + saline group than in the sham + acid group (*P* < 0.01). c-fos expression was much higher in the AM in the operation + acid group than in the operation + saline group (*P* < 0.01) (*n* = 8, ^∗∗^*P* < 0.01).

To evaluate morphological control of DMV destruction, the animals were deeply anesthetized with urethane (1.5 g/kg i.p.). Then, the animals were transcardially perfused with 9 g/L of saline, followed by 40 g/L of paraformaldehyde in 0.1 mol/L PBS (pH 7.3), and their brains were removed and placed into 40 g/L of paraformaldehyde in 0.1 mol/L phosphate-buffered saline (PBS, pH 7.3). The area of DMV destruction was evaluated on serial sections stained with HE. The paraffin sections showed that the lesions were located 0.15 mm below the fourth ventricle and 0.5 mm-1 mm on the right side (Figures 1(a) and 1(b)), indicating that the DMV was located accurately.

The rats were free to eat and drink after the operation. Two weeks later, the esophagus was perfused with acid or saline solution.

### 2.4. Esophageal Perfusion Approach

After the rat was completely anesthetized, the abdominal and gastric walls were incised, and a drainage cannula was inserted into the gastric cardia to collect run-off solution from the esophagus. The anesthetized rat was strapped supine to an animal board and then positioned with its head elevated at a slight angle (20-30°). A single lumen clear vinyl tube (ID 0.3 mm, OD 0.5 mm) was passed through the mouth and into the esophagus. The tip of the tube was located 2 cm above the esophagogastric junction. Then, the tube was connected to a continuous perfusion pump (Medical Equipment Co. Ltd., Zhejiang University, Hangzhou, China). A solution (pH 1.5) containing hydrochloric acid (0.1 mol/L HCl) and pepsin (3000 U/mL, Sigma, USA) was perfused continuously at a rate of 20 mL/h for 90 min. Saline was used as a control [13].

After esophageal perfusion, the rat was left undisturbed for 60 min before being deeply anesthetized with urethane (1.5 g/kg i.p.). Then, the animal was transcardially perfused with 9 g/L of saline, followed by 40 g/L of paraformaldehyde in 0.1 mol/L PBS (pH 7.3).

Prior to perfusion of animals, a horizontal section was taken from the lower part of the esophagus and processed with iced saline solution. A piece of the esophageal tissue was fixed in 10% formaldehyde solution for 24 h and then embedded in paraffin, sectioned at a 4 *μ*m thickness, and stained with HE. Slices were also taken to assess NF-*κ*B p65 immunoreactivity. Each specimen was examined by a light microscope to detect signs of inflammatory changes. The extent of microscopic mucosal damage was determined and graded by two independent observers who were unaware of the treatment given. The scoring criteria for esophagitis were modified in the past literature [14] as follows: epithelial changes (basal hyperplasia, mitosis, papillomatosis, epithelial splitting, erosion, and ulceration), maximal score 40; inflammation (intraepithelial leukocytes, intensity, and extension), maximal score 40; and vascular alterations (edema, congestion, and hemorrhage), maximal score 20. The remaining esophageal tissue was preserved in liquid nitrogen. After the specimen collection was completed, the samples were homogenized using polytron in saline solution (pH 7.4) prior to the examination. The homogenates were centrifuged at 3000 r/min for 15 min at 4°C, and the resulting supernatants were used for TNF-*α*, IL-6, IL-1*β*, and PGE2 analyses by ELISA (R&D Systems, USA).

### 2.5. Immunohistochemistry Staining

After perfusion of animals, the brains were removed, postfixed in the same fixative at 4°C for 18–20 h, and paraffin-embedded prior to analysis. We performed the trimming of the brain using a brain matrix (68709, Shenzhen Ryward Life Technology Co. Ltd). According to the research of Defazio et al. [15], we chose the seventh channel (at the optic chiasm level, corresponding to −1.8 mm from the Bregma) for the AM, PVN, and SON; the fifteenth channel (corresponding to −9.8 mm from the Bregma) for the PBN; and the nineteenth channel (corresponding to −13.8 mm from the Bregma) for the NTS/DMV, RNM, RA, and AP. Coronal sections (4 *μ*m) of the brain were cut with a Leica RM2235 microtome (Leica Microsystems, Germany).

The paraffin sections were deparaffinized before processing. The antigen was retrieved by microwaving. Brain sections were incubated with c-fos (diluted 1:800; SC-52, Santa Cruz, USA) for 75 min. Esophageal sections were incubated with NF-*κ*B p65 (diluted 1:400; 8242, Cell Signaling Technology, USA). After washing with PBS, the sections were incubated with a biotinylated secondary antibody (Zhongshan Jinqiao, China) for 13 min. All incubation steps were performed at room temperature. PBS was used as a negative antibody control. Immunohistochemistry staining was visualized with microscope, photographed with a Hamamatsu NanoZoomer 2.0 HT slice scanning digital device, and the average positive-stained cells were calculated with Image-Pro Plus 6.0 [16]. Cell counting was performed by two independent observers, blinded to the experimental groups.

The neurons of c-fos-positive cells had a red nucleus. The borders of the nucleus were determined according to the stereotaxic atlas by Paxinos and Watson [16, 17]. To avoid the error caused by the slight difference in cut planes of the same nucleus, each nucleus was calculated based on five consecutive sections [17]. Data were expressed as number of c-fos-positive neurons [18].

NF-*κ*B p65-positive cells in the esophagus had a dark brown nucleus. It means that the nucleus stained more strongly than the cytoplasm did [19]. For each section, three different areas equivalent to the magnification of ×200 in the electronic slice were selected to calculate the average positive-stained cells [20].

### 2.6. Statistical Analysis

GraphPad Prism 5.1 was used for data analysis. The data were expressed as mean ± SE of the respective brain areas. Groups were compared by one-way ANOVA with a post hoc Tukey's test. *P* < 0.05 was considered significant.

## 3. Results

### 3.1. Esophageal Instillation and DMV Destruction Upregulated TNF-*α*, IL-1*β*, IL-6, and PGE2 in Esophageal Tissue

**Figure 2:**
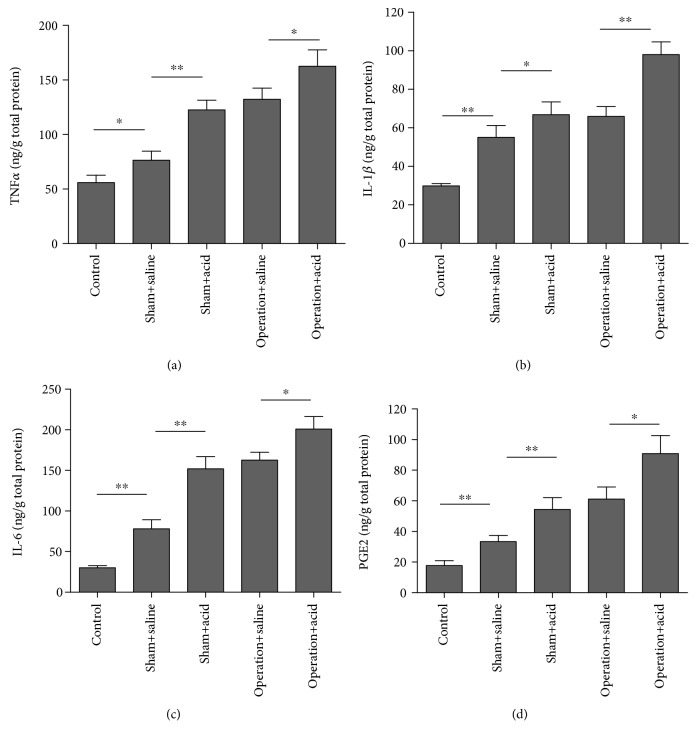
DMV destruction aggravated proinflammatory cytokine expression in an esophageal instillation model. The concentration of the cytokines TNF-*α* (a), IL-1*β* (b), IL-6 (c), and PGE2 (d) in esophageal tissue in experimental groups more significantly increased than in the control group (*n* = 8, ∗*P* < 0.05 and ∗∗*P* < 0.01). Specifically, TNF-*α*, IL-1*β*, IL-6, and PGE2 expression more significantly increased in the esophageal tissues of the operation + acid groups compared to those of the operation + saline groups (*n* = 8, ^∗^*P* < 0.05 and ^∗∗^*P* < 0.01).

As shown in Figures 2(a)–2(d), TNF-*α*, IL-1*β*, IL-6, and PGE2 expression in experimental groups increased more significantly than that in the control group. Specifically, TNF-*α*, IL-1*β*, IL-6, and PGE2 expression increased more in the esophageal tissues of the operation + acid groups compared to the tissues of the operation + saline group (*P* < 0.05).

### 3.2. Esophageal Instillation and DMV Destruction Led to Severe Esophageal Tissue Damage in Rats

**Figure 3:**
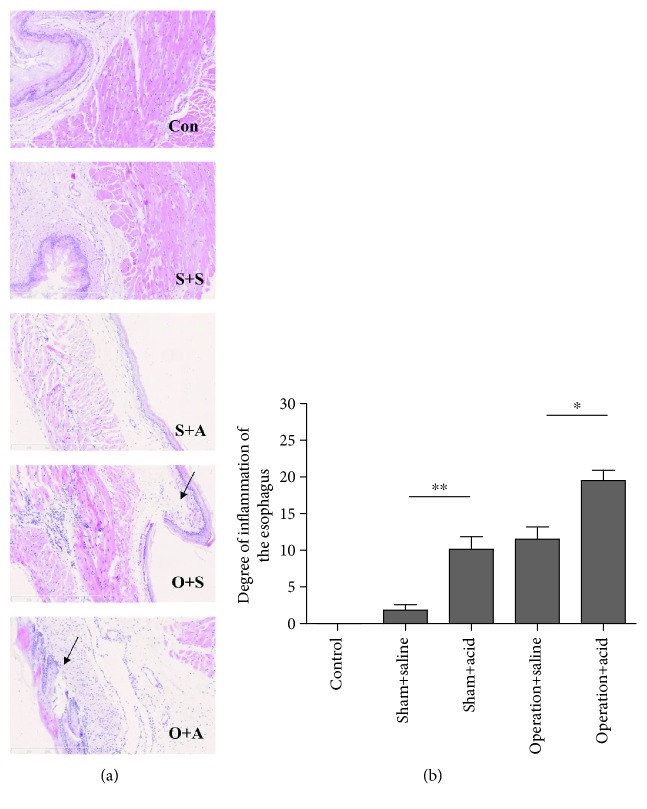
Esophageal instillation and DMV destruction led to severe esophageal tissue damage in rats. (a) HE staining of the lower esophagus in the control group (Con), sham + saline group (S+S), sham + acid group (S+A), operation + saline group (O+S), and operation + acid group (O+A). Arrows shows submucosal edema. (b) The esophageal tissue damage scores under the light microscope in rats in the acute esophagitis model induced by esophageal instillation and DMV destruction (*n* = 8, ^∗^*P* < 0.05 and ^∗∗^*P* < 0.01). The microscopic esophagitis scores in the lower esophageal segment were significantly higher in the sham + acid group than in the sham + saline group (1.83 ± 0.75) (*P* < 0.01). No significant difference in the microscopic esophagitis scores was observed between the operation + saline group (11.5 ± 1.69) and the sham + acid group (10.13 ± 1.73) (*P* > 0.05). The microscopic esophagitis scores were higher in the lower esophageal segment of the operation + acid group (19.5 ± 1.41) than in the operation + saline group (*P* < 0.05).

The microscopic esophagitis scores in the lower esophageal segment were significantly higher in the sham + acid group than in the sham + saline group (1.83 ± 0.75) (*P* < 0.01) (Figure 3(b)). No significant difference in the microscopic esophagitis scores was observed between the operation + saline group (11.5 ± 1.69) and the sham + acid group (10.13 ± 1.73) (*P* > 0.05) (Figure 3(b)). However, the microscopic esophagitis scores were much higher in the lower esophageal segment of the operation + acid group (19.5 ± 1.41) than in the operation + saline group (*P* < 0.05) (Figure 3(b)).

### 3.3. Esophageal Instillation and DMV Destruction Caused Activation of NF-*κ*B in Esophageal Tissue of Rats

**Figure 4:**
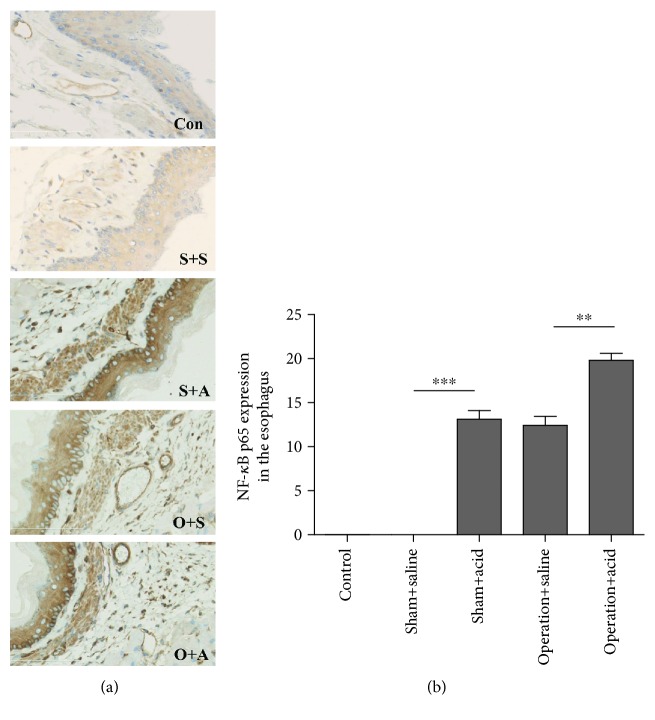
Esophageal instillation and DMV destruction caused activation of NF-*κ*B in esophageal tissue of rats. (a) NF-*κ*B p65-positive cells with dark brown nuclei in the esophagus of all animals. Con: control group (×400); S+S: sham + saline group (×400); S+A: sham + acid group (×400); O+S: operation + saline group (×400); O+A: operation + acid group (×400). (b) The activation of NF-*κ*B p65 in the lower esophageal segment increased more significantly in the operation + acid group than in the operation + saline group (*P* < 0.01). No significant difference in the NF-*κ*B p65 activation in the esophagus was observed between the operation + saline group and the sham + acid group (*P* > 0.05). In the control and sham + saline rats, NF-*κ*B p65 activation was not observed (*n* = 8, ^∗∗^*P* < 0.01).

NF-*κ*B p65 activation was observed in the nucleus of inflammatory cells in the lamina propria and the submucosa of the esophageal mucosa. The activation of NF-*κ*B p65 in the lower esophageal segment was significantly higher in the operation + acid group (19.6 ± 0.87) than in the operation + saline group (12.4 ± 0.94) (*P* < 0.01) (Figure 4(b)). No significant difference in NF-*κ*B p65 activation in the esophagus was found between the operation + saline group (12.4 ± 0.94) and the sham + acid group (13.0 ± 1.03) (*P* > 0.05) (Figure 4(b)). In the control and the sham + saline rats, NF-*κ*B p65 activation was not observed (Figure 4(b)).

### 3.4. DMV Destruction Attenuated c-fos Expression in the AM, PVN, NTS/DMV, RNM, and AP

**Figure 5:**
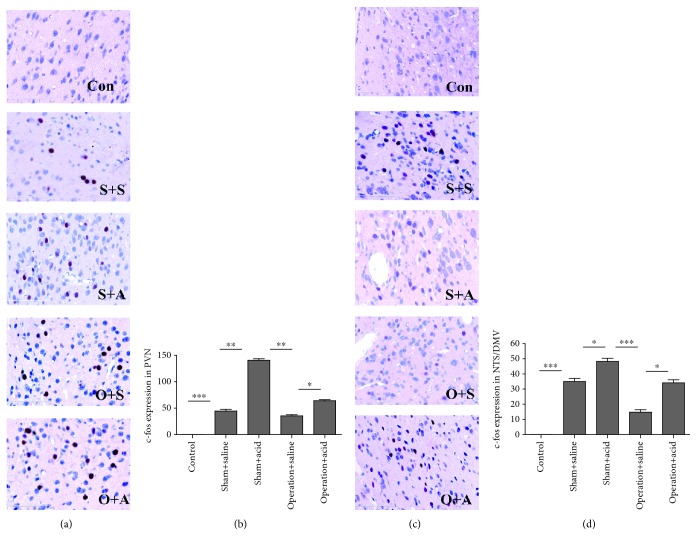
DMV destruction downregulated c-fos expression in the PVN and the NTS/DMV. (a) c-fos-positive cells with red nuclei in the PVN in the different groups. (×400). (b) Induction of c-fos expression in the PVN by esophageal instillation and DMV destruction. c-fos expression in the PVN increased more significantly in the sham + acid group than in the sham + saline group (*P* < 0.01). After DMV destruction, c-fos expression more significantly decreased in the PVN in the operation + saline group than in the sham + acid group (*P* < 0.01). c-fos expression was higher in the PVN in the operation + acid group than in the operation + saline group (*P* < 0.05). (c) c-fos-positive cells with red nuclei in the NTS/DMV in the different groups (×400). (d) Induction of c-fos expression in the NTS/DMV by esophageal instillation and DMV destruction. c-fos expression in the NTS/DMV increased more in the sham + acid group than in the sham + saline group (*P* < 0.05). After DMV destruction, c-fos expression more significantly decreased in the NTS/DMV in the operation + saline group than in the sham + acid group (*P* < 0.001). c-fos expression was higher in the NTS/DMV in the operation + acid group than in the operation + saline group (*P* < 0.05) (Con: control group; S+S: sham + saline group; S+A: sham + acid group; O+S: operation + saline group; O+A: operation + acid group. *n* = 8, ^∗^*P* < 0.05, ^∗∗^*P* < 0.01, ^∗∗∗^*P* < 0.001).

**Figure 6:**
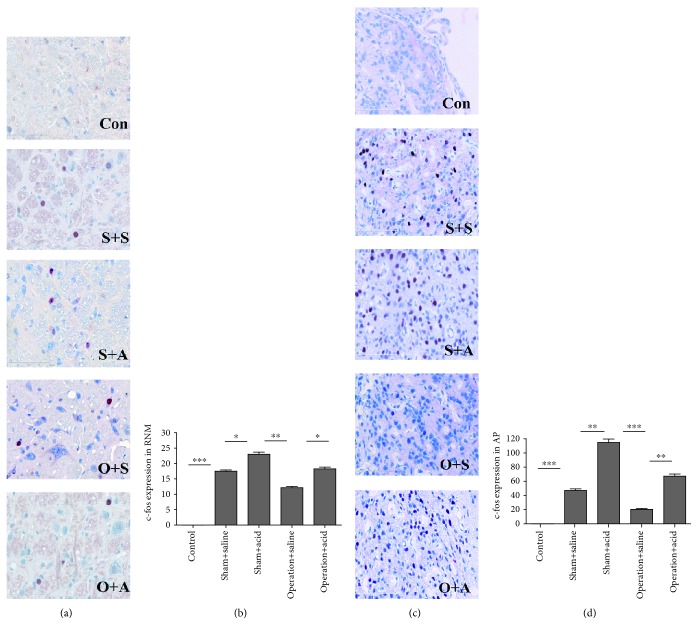
DMV destruction attenuated c-fos expression in the RNM and AP. (a) c-fos-positive cells with red nuclei in the RNM in the different groups (×400). (b) Induction of c-fos expression in the RNM by esophageal instillation and DMV destruction. c-fos expression in the RNM increased more in the sham + acid group than in the sham + saline group (*P* < 0.05). After DMV destruction, c-fos expression decreased more significantly in the RNM in the operation + saline group than in the sham + acid group (*P* < 0.01). c-fos expression was higher in the RNM in the operation + acid group than in the operation + saline group (*P* < 0.05). (c) c-fos-positive cells with red nuclei in the AP in all groups (×400). (d) The effects on c-fos expression in the AP of esophageal instillation and DMV destruction. c-fos expression in the AP increased more significantly in the sham + acid group than in the sham + saline group (*P* < 0.01). After DMV destruction, c-fos expression more significantly decreased in the AP in the operation + saline group than in the sham + acid group (*P* < 0.001). c-fos expression was much higher in the AP in the operation + acid group than in the operation + saline group (*P* < 0.01) (Con: control group; S+S: sham + saline group; S+A: sham + acid group; O+S: operation + saline group; O+A: operation + acid group. *n* = 8, ^∗^*P* < 0.05, ^∗∗^*P* < 0.01, and ^∗∗∗^*P* < 0.001).

In the control rats without any stimulation, c-fos expression was observed occasionally in the AM and PBN. In all other groups, c-fos expression was obviously present in the AM, PVN, SON, PBN, NTS / DMV, NA, RNM, and AP. Compared with the sham + saline group, c-fos expression increased more in the AM, PVN, PBN, NTS / DMV, NA, RNM, and AP in the sham + acid group (*P* < 0.05) (Figures 1(d), 5(b), 5(d), 6(b), and 6(d); the PBN and the NA were not shown). However, no significant difference in c-fos expression in the SON was observed between the sham + saline group and the sham +acid group (*P* > 0.05) (data not shown).

After DMV destruction, c-fos expression of operation groups decreased in the AM, PVN, PBN, NTS /DMV, RNM, NA, and AP, especially in the AM, PVN, NTS/DMV, RNM, and AP (*P* < 0.05) (Figures 1(d), 5(b), 5(d), 6(b), and 6(d)). c-fos expression was much higher in the AM, PVN, PBN, NTS/DMV, RNM, NA, and AP in the operation + acid group than that in the operation + saline group (*P* < 0.05) (Figures 1(d), 5(b), 5(d), 6(b), and 6(d); the PBN and the NA were not shown).

No significant difference in c-fos expression was observed in the bilateral nerve nucleus except NTS /DMV (*P* > 0.05). After the induction of unilateral DMV damage, c-fos expression in the NTS/DMV was calculated using the contralateral nerve nucleus.

## 4. Discussion

The pathogenesis of reflux esophagitis is associated with oxidative stress, inflammation, and apoptosis [21]. Previous research has demonstrated that the TNF-*α*, IL-1*β*, and IL-6 concentrations increased in the esophageal tissues of rats in several reflux esophagitis models [22, 23]. In our study, the concentrations of inflammatory cytokines in esophageal tissues, including TNF-*α*, IL-1*β*, and IL-6, were significantly higher in the operation + acid group than those in the sham + saline groups.

The aforementioned inflammatory cytokines are important in microbial infection and tissue damage. TNF-*α* is a major cytokine which is released by monocytes and macrophages and induces inflammation and cytotoxicity [24]. In the present study, we found that the lesions of the lower esophageal segment were more serious in the operation + acid group than in the other groups in the rats. An obvious mucosal damage was observed in the operation + acid group, including epithelial basal cell hyperplasia, papillary hyperplasia, and inflammatory cell infiltration. Previous data indicated that TNF-*α* and IL-1*β* might activate microvascular endothelial cells and lead to increased PGE2 production [25]. Thus, the above procedure may aggravate esophageal inflammation.

NF-*κ*B activation is important in cellular inflammation. The NF-*κ*B pathway can be triggered by inflammatory cytokines (such as TNF-*α* or IL-1*β*). It is normally predominantly located in the cytoplasm but transferred to the nucleus upon activation [26]. We found that the normal and the sham + saline esophagus of rats have no detectable active NF-*κ*B p65, while high levels of active NF-*κ*B p65 were found in the operation + acid group. NF-*κ*B is an important factor contributing to reflux esophagitis though upregulating its downstream target gene expressions involved in inflammation. The inflammatory cytokines, including TNF and IL-1*β*, may also start a feedback loop for a second stage of NF-*κ*B activation, resulting in severe esophageal tissue damage in rats [27].

Previous research showed that the vagus efferent nerve released Ach through intracellular signal transduction, which could inhibit the synthesis and release of the inflammatory factors TNF-*α*, IL-6, and IL-1*β* by macrophages after endotoxin stimulation [28]. Macrophages are reported to play an important role in the CAP [29, 30].

Recent researches have revealed the anti-inflammatory function of the vagus nerve. According to the above studies, the anti-inflammatory function was mediated through several pathways, some of which were still under debate [28, 31]. The first one was the hypothalamic–pituitary–adrenal (HPA) axis which could release cortisol stimulated by vagal afferent fibres. The second one was the CAP, which could release Ach through vagal efferent fibres. Ach binds to *α*-7-nicotinic Ach receptors of the enteric neuron synaptic junction with macrophages to inhibit the release of proinflammatory cytokine TNF-*α*. The last pathway was the splenic sympathetic anti-inflammatory pathway, which could release norepinephrine by the splenic sympathetic nerve. Norepinephrine binds to the *β*2 adrenergic receptor of splenic lymphocytes that release Ach. Similarly, Ach binds to *α*-7-nicotinic Ach receptors of spleen macrophages to inhibit the release of proinflammatory cytokine TNF-*α*.

Vagal efferent fibres originate from the DMV. In order to reveal the role of the CAP in acute esophagitis of a rat model, unilateral DMV was damaged. In rats, the vagus nerve innervates all of the digestive tracts except for the rectum [31]. We found that after unilateral DMV damage, the protective function of the vagus efferent nerve by Ach attenuated, esophageal inflammation aggravated, and the concentrations of the proinflammatory cytokines TNF-*α*, IL-6, and IL-1*β* significantly increased.

In the present study, c-fos expression was observed occasionally at very low expression levels only in the AM and PBN in the control rats without any stimulation. c-fos expression in the AM, PVN, PBN, NTS/DMV, NA, RNM, and AP increased significantly in the acid group compared with the saline group, suggesting that esophageal acid stimulation activated these nerve nuclei [10, 32].

Acid perfusion to the cervical esophagus significantly activated the DMV, NA, AP, and PBN and all subnuclei of the NTS but not the ventral subnucleus of NTS [33]. On the other hand, acid perfusion to the thoracic esophagus activated neurons in only a few of the NTS subnuclei and the DMV, while in this study, NTS, DMV, NA, AP, and PBN were all activated when the acid-pepsin infusion tube was located 2 cm above the esophagogastric junction. We found that the infusion to the thoracic esophagus inevitably caused accumulation of fluid in the pharynx and brief periods of aspiration. So we concluded that it is highly likely that the acid-pepsin infusion was refluxed to the upper esophagus and pharynx.

The vagal afferent nerve initiates from the mucosa and transmits to the muscle layers of the digestive tract, conveying the information to the NTS and the AP. After that, visceral information is sent to the forebrain areas such as the amygdala via a relay through the PBN and the HPA axis. Particularly, the vagal afferent nerve could relay information to the NTS and then project information to the PVN [31]. In summary, the activation of the NTS, DMV, AP, NA, PBN, AM, and PVN by acid perfusion to the esophagus is caused by the vagal afferent nerve. After the DMV destruction, c-fos expression was reduced in the AM, PVN, PBN, NTS/DMV, RNM, NA, and AP, especially in the AM, PVN, NTS/DMV, RNM, and AP. The DMV is a motor nucleus instead of a sensory nucleus. Thus, it is difficult to explain how lesions of the DMV could alter c-fos levels of the brain nucleus of rostral DMV. One possible explanation might be that the DMV efferents altered various digestive tract organs and perhaps decreased motility, which decreased vagal afferent activity and thus decreased c-fos expression of the rostral brain nucleus. If we inadvertently had lesioned the NTS, it would have blocked vagal afferent excitation of more rostral nuclei. However, the DMV lesion was found to located accurately when being evaluated on paraffin sections. Therefore, further investigation should be conducted in this aspect in the future.

The role of the CAP was confirmed in endotoxemia and ischemic and hemorrhagic stroke [34, 35], but its role in regulation in an acute model of esophagitis is still not clear. The use of DMV destruction to study the protective effects of the CAP in an acute model of esophagitis in rats has not been reported previously. The present findings suggest that the CAP has a protective effect on the acute esophagitis rat model and it provides a new therapeutic alternative for RE. Proton pump inhibitor (PPI) treatments act downstream of the RE. Hence, treatments acting upstream would be worth exploring. The device of the transcutaneous vegetative nervous system was accepted for epilepsy [36] and could be used for inflammatory digestive disorders in the future. The use of neuromodulation by bioelectronic devices could be an alternative nondrug therapy for RE or could be combined with PPI treatments.

## 5. Conclusion

In conclusion, in this study we successfully established an acute esophagitis model in rats by unilateral DMV destruction and esophageal acid instillation and subsequently found that the DMV lesion can aggravate esophageal inflammation and injury in the rat model. These data provide experimental and theoretical evidence to support the use of neuromodulation as a treatment for RE.

## Data Availability

The data used to support the findings of this study are included within the article.
